# The *Homo erectus* Female Revisited

**DOI:** 10.1002/ajhb.70091

**Published:** 2025-06-24

**Authors:** Leslie C. Aiello

**Affiliations:** ^1^ Wenner‐Gren Foundation for Anthropological Research New York New York USA

**Keywords:** body size, energetics, *Homo erectus*, human evolution, reproduction

## Abstract

The Energetic Consequences of Being a *Homo erectus* Female was published in the *American Journal of Human Biology* over two decades ago. This paper drew attention to the high body‐size‐related reproductive costs of an *H. erectus* female if she retained the same reproductive schedule of smaller‐bodied earlier hominins modeled on the schedule for modern *Pan*. The main conclusion was that the energetic cost per offspring would be significantly reduced by adopting a modern human reproductive schedule with a shorter lactation period and an overall shorter interbirth interval. To make this possible and support the energetic requirements of the larger body size, there would have had to be a fundamental shift in subsistence behavior involving a higher‐quality diet and intergenerational cooperation in food acquisition. This paper re‐evaluates these conclusions based on recent energetic research developments. Although the modeling parameters have changed, the conclusions are still valid. Their implications are discussed in light of modern research on the increase in body and brain size and the evolution of cooperative subsistence behavior.

## 1 Introduction

In 2002, Cathy Key and I published the Energetic Consequences of Being a *Homo erectus* Female (Aiello and Key 2002). This followed on from my interests in the energetics of brain evolution (Aiello and Wheeler 1995) and her interests in cooperation, paternal care, energetics, and the evolution of hominid social groups (Key and Ross 1999; Key 1998). In the *H. erectus* female paper, we explored the energetic implications of body size increase and sexual dimorphism decrease between the australopithecines and later *Homo*, particularly *H. erectus* and *H. sapiens* . We focused on the energy requirements of gestation and lactation in these hominins. Our energetic modeling was based on activity patterns, body mass, and the life histories of living primates. We assumed that the australopiths had a reproductive schedule like that of living chimpanzees (*Pan*) and *that H. erectus* had a pattern like that *of* humans (Table 1).

**TABLE 1:** Reproductive schedules for *Pan* and *H. sapiens* .

	Gestation^a^	Lactation	Cycling	Interbirth interval
*Pan troglodytes*	235	1464	314	2013
*H. sapiens*	267	764	83	1114

^a^ Reproductive schedule in days.

*Source:* Data from Aiello and Key (2002).

Larger bodies are energetically more costly than smaller bodies, and we assumed that the cost of both gestation and lactation scales with body mass. Our main conclusion was that if *H. erectus* had similar reproductive schedules to the australopiths (gestation and lactation lengths and interbirth intervals modeled as *Pan*), the daily energetic requirements for *H. erectus* females would have been significantly higher than for smaller‐bodied australopith females, as would the total cost per offspring. This is due to the larger body size of *H. erectus* . However, adopting a human reproductive schedule with a shorter period of lactation and a shorter interbirth interval would significantly reduce the energetic cost per infant and, simultaneously, result in the possibility of more offspring per female over her reproductive life. The downside would be the higher daily body‐size‐related cost to the larger female *H. erectus*, together with the added burden of providing resources for sequential dependent offspring. These offspring would, out of necessity, be weaned before they were mature enough to provide their own food independently.

We concluded that this scenario would only be viable if there were an economic division of labor, whereby the mother (and her increased energy requirements) was supported by other individuals such as older related females, males, and possibly older children. We also suggested that these body‐size‐related constraints on reproduction may have formed the basis for modern human life history patterns and social organization based on cooperation and economic division of labor. This was not a novel conclusion and followed on from earlier work on the importance of cooperation in human life‐history evolution (Hawkes et al. 1998; Hrdy 1999; Kaplan et al. 2000). The novel aspect of our *H. erectus* female paper was the attempt to quantify the issue through energetics.

Over the more than two decades since this paper was published, considerable research has been done on human energetics and the determinants of modern human cooperative social organization. This commentary aims to (1) reassess our modeling in the context of contemporary research on human energetics and (2) review some of the significant recent milestones related to the evolution of human life history patterns and cooperation‐based social organization. The main question is whether the conclusions of the *H. erectus female* paper are still valid and significant.

## 2 Modeling the Energetics of the *Homo erectus* Female

There has been a revolution in human energetics resulting from the ability to measure the total energy expenditure (TEE)^1^ directly for humans and non‐human primates using the doubly labeled water (DLW) technique (Speakman 1997). This technique has become the gold standard for energetic measurement, eliminating the guesswork involved in the old additive methodology. It involves the subject drinking water labeled with two stable isotopes, deuterium (^2^H) and oxygen‐18 (^18^O). Deuterium is eliminated from the body through water loss (e.g., sweat, urine, etc.) and oxygen‐18 through water loss and carbon dioxide production. Analyzing the ratio between these two stable isotopes enables the accurate estimation of TEE.

The surprising result is that the old additive TEE predictive equations based on basal metabolic rate and activity budget are essentially wrong (Pontzer 2017a). The new DLW technique reveals grade differences that separate humans from the great apes and the great apes from each other. Humans have the highest TEE for their body size, followed by *Pan*, Gorilla, and *Pongo*, which has the lowest TEE (Pontzer et al. 2016). The realization that there are species‐specific TEEs is part of the data that led to the development of the Constrained Energy Hypothesis, which suggests that an organism's total energy budget is selected for and maintained within a relatively narrow range (Pontzer 2015, 2017a; Pontzer et al. 2016).

**FIGURE 1:**
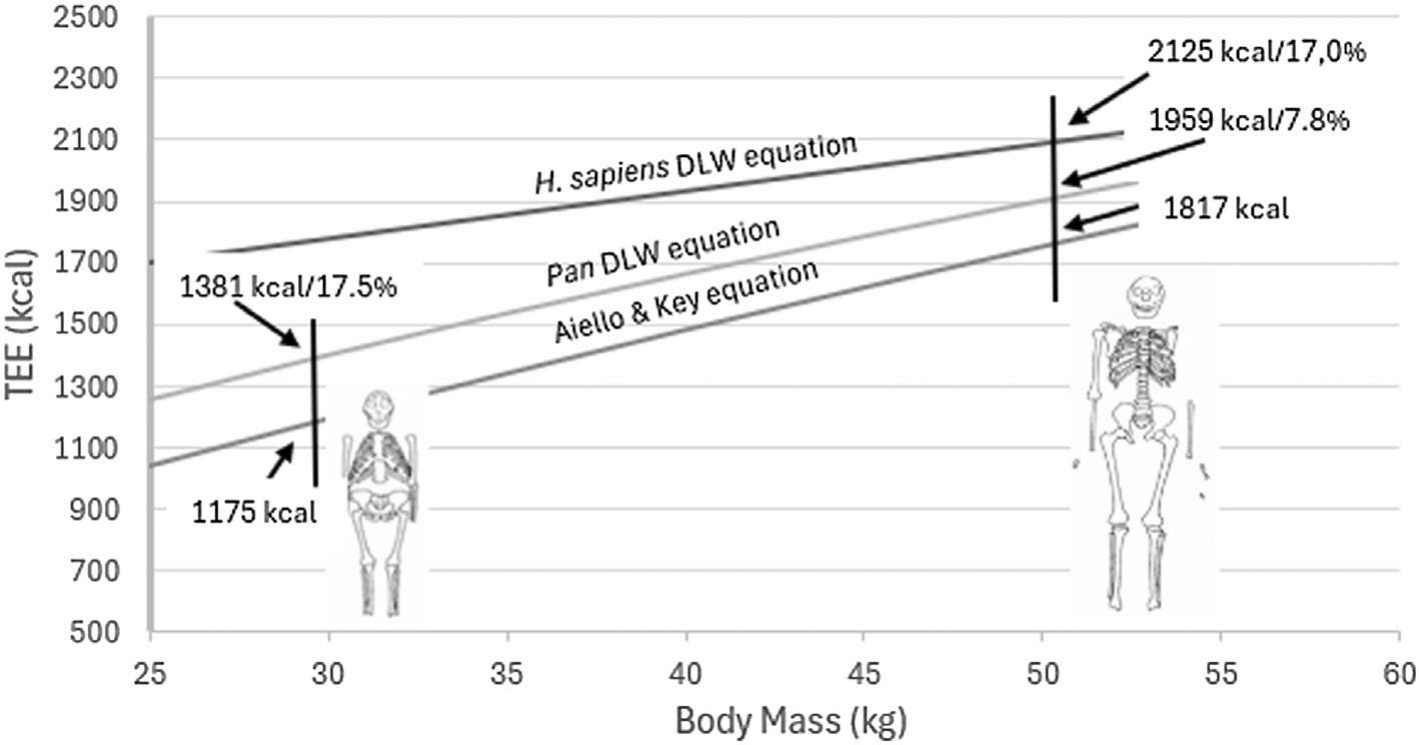
Equations for the prediction of TEE (kcal). Aiello and Key (2002) equation: TEE = 10^(LOG10(Body Mass) × 0.75 + 1.97)^. *Pan* equation (Kraft et al. 2021): Ln(TEE) = 0.602 × ln(BM) + 0.792. *H. sapiens* equation (Pontzer et al. 2016): TEE = 0.065 × Mass − 0.025 × Age + 5.984. Body mass (kg) for *A. afarensis* = 29.3 kg and for *H. erectus* = 52.4 kg (Aiello and Key 2002). Age = 20 years. Percentages = percentage increase over the Aiello and Key (2002) predictions. Mcal = megacalories. *Source:* Diagrams from Ruff (1993).

The grade differences in predicted TEE for humans and apes have significant consequences for the modeling in Aiello and Key (A&K) (2002). Both the human and *Pan* DLW‐predicted TEEs are significantly higher than those predicted by the equation used in A&K (2002), which was based on the activity budget TEE model developed by Key and Ross (Key and Ross 1999) (Figure 1). The DWL equation for *Pan* (Kraft et al. 2021) results in a 17.5% higher body‐size estimated TEE for a 29.3 kg *A. afarensis*. For a 52.3 kg *H. erectus* , the human equation (Pontzer et al. 2016) results in a 17% higher TEE, while the *Pan* equation shows a 7.8% increase for *H. erectus* .

This increase in predicted TEE also affects the predictions for gestation and lactation energy costs. The original A&K estimates for gestation and lactation costs are modeled as proportions of the predicted A&K TEE, again based on Key and Ross (1999). Gestation was assumed to increase daily TEE by 25% and lactation by 39% for *A. afarensis* and 37% for *H. erectus* (because of a modest allometric effect). If these costs are averaged over the interbirth interval, the total cost of reproduction (gestation plus lactation) would be between 23% and 24% of total TEE for both *A. afarensis* (modeled on the *Pan* reproductive schedule) and *H. erectus* (modeled on the human reproductive schedule). The daily reproductive cost would be 557 kcal/day for *H. erectus* and 368 kcal/day for *A. afarensis* (Table 2).

**TABLE 2:** Reproductive costs.

	Gestation (kcal/%TEE)	Lactation (kcal/%TEE)	Reproduction (kcal/%TEE)	Interbirth interval	Daily reproduction
Aiello and Key (2002) model					
*H. erectus*	121,151/4.6%	499,198/18.9%	620,349/23.5%	1114	557
*A. afarensis*	69,031/2.2%	670,878/21.6%	739,909/23.8%	2013	368
DLW model					
*H. erectus*	66,694/2.8%	271,505/11.5%	338,198/14.3%	1114	304
*A. afarensis*	34,463/1.2%	262,916/9.5%	297,379/10.7%	2013	148

*Note:* Reproductive costs are based on daily gestation and lactation costs (Figure 2) summed over the gestation and lactation periods from the schedule in Table 1. Reproduction (kcal) = the summed costs of gestation and lactation. %TEE = the reproduction costs as a percentage of the TEE for the total interbirth interval. Daily Reproduction = the daily cost (kcal) of reproduction averaged over the interbirth interval. The format of this table is comparable to (Pontzer et al. 2016, tab. S2). Note that the *daily* gestation costs for both *A. afarensis* and *H. erectus* under the Aiello and Key modeling = 25% of daily TEE, but the total cost of gestation is only 2.2% of the total TEE for the interbirth interval for *A. afarensis* and 4.6% for *H. erectus* based on the reproductive schedule in Table 1. The same is true for lactation, which is 39% of daily TEE for *A. afarensis*, but 21.6% of total TEE for the interbirth interval, and 36% of daily TEE for *H. erectus* , but 18.9% of total TEE for the interbirth interval.

The DLW estimates for gestation and lactation for humans and *Pan* (Table 2) are based on (Pontzer et al. 2016) and are in line with DLW costs estimated in the NAS report on Dietary Reference Intakes for Energy (National Academies of Sciences, 2023). Pontzer et al. (2016) suggest that human costs for gestation and lactation combined would be 14.3% of TEE, averaged over the interbirth interval. Scaled to human gestation and lactation costs, *Pan* costs would be 10.7% of TEE (Pontzer et al. 2016). These are significantly less than the A&K modeled percentages.

To be comparable to the A&K estimates, the reproductive costs for *Pan* (10.7% of TEE) and humans (14.3% of TEE) were used together with the DLW TEE equations to predict the reproductive costs of *A. afarensis* and *H. erectus* (Table 2). The results show that the daily reproductive cost over a single reproductive event for an *H. erectus* modeled as a human would be 54.5% of that predicted by A&K (304 vs. 557 kcal/day), and for *A. afarensis* modeled as *Pan*, 40.2% of the A&K prediction, (148 vs. 368 kcal/day).

Therefore, the DLW results produce TEE predictions that are higher than the A&K predictions, and the new modeling for human and *Pan* gestation and lactation produce costs that are lower. In addition, the A&K modeling is additive. Total TEE during the reproductive cycle is the predicted A&K TEE plus the gestation and lactation TEE. The DLW TEE results include the cost of both gestation and lactation. Under the Constrained Energy Hypothesis, these costs would be balanced against other energy demands to maintain the total energy budget within the constraints for each species.

**FIGURE 2:**
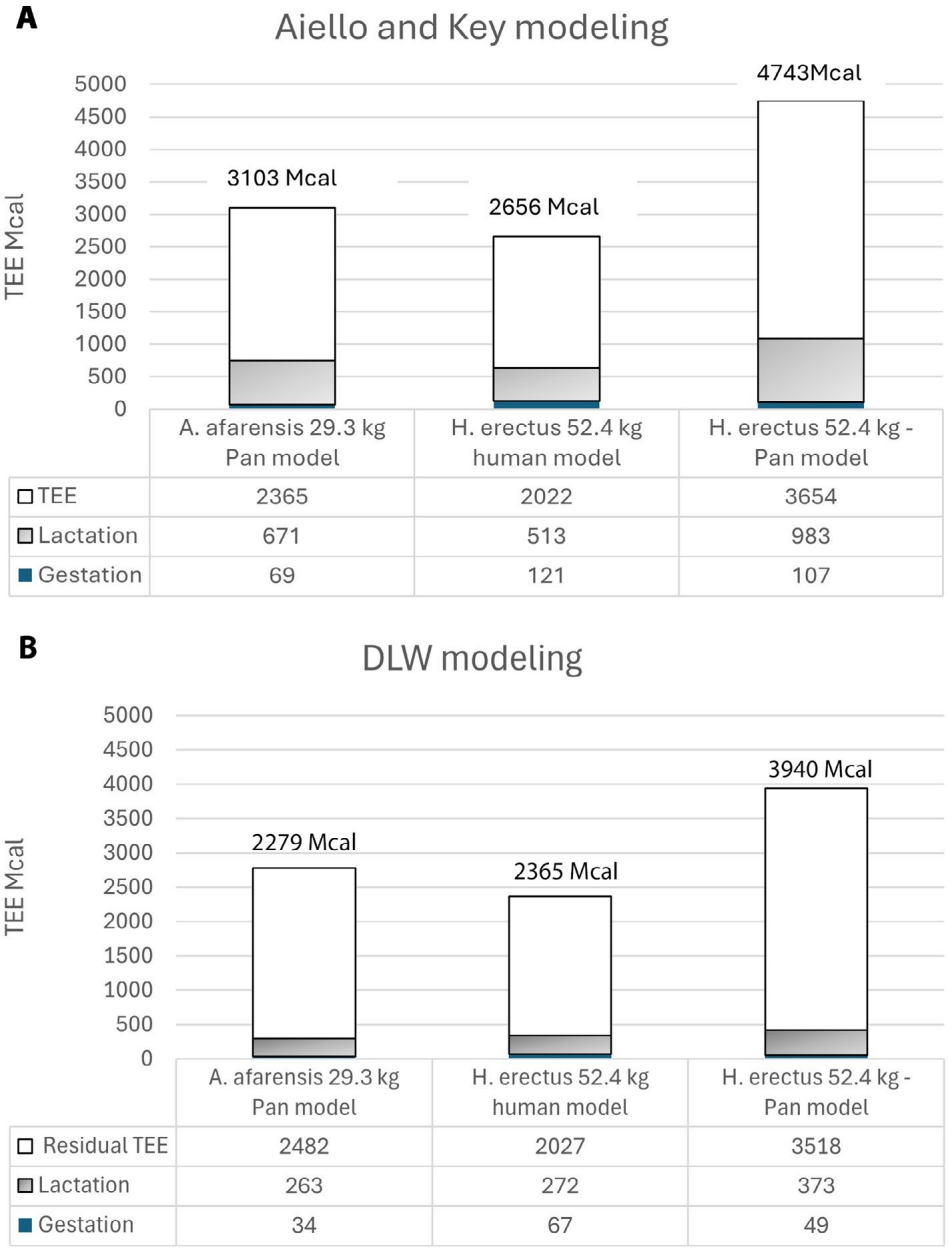
Total TEE for a single reproductive event based on the reproductive schedule in Table 1. TEE in Mcal. (A) Original Aiello and Key modeling based on the TEE equation in Figure 1, daily gestation = TEE × 25%, daily lactation = TEE × 39% for *A. afarensis* and TEE × 36% for *H. erectus* . Summed over the entire interbirth interval, total cost of gestation for *A. afarensis* = 2.2% total TEE for the interbirth interval, and *H. erectus* = 4.6%. The total cost of lactation for *A. afarensis* = 21.6% of total interbirth TEE, and *H. erectus* = 18.9%. (B) DLW modeling based on TEE equations in Figure 1 for humans and *Pan*. Gestation = 1.2% TEE for *A. afarensis* modeled as *Pan* and 2.8% TEE for *H. erectus* modeled as a human. Lactation = 9.5% TEE for *A. afarensis* modeled as *Pan* and 11.5% TEE for *H. erectus* modeled as a human. Percentages are based on the data in Pontzer et al. (2016), tab. S2). Note that the Aiello and Key modeling is additive; the gestation and lactation costs are added to the daily TEE. The DLW daily TEE includes the gestation and lactation costs. The “residual TEE” in B is the daily TEE minus the gestation and lactation costs. The figures above each bar chart are the total costs per reproductive event. Reproductive schedules from Aiello and Key (2002) and Table 1. Total reproductive costs under both the Aiello and Key and DLW modeling are from Table 2.

How does this affect A&K's general conclusion that adopting a human reproductive schedule by a larger‐bodied *H. erectus* reduces the overall energy cost of each reproductive event? The answer is not much (Figure 2). In the A&K model, the cost of a single reproductive event for a larger‐bodied *H. erectus* modeled as a human would be 86% of the cost of a much smaller‐bodied *A. afarensis* modeled as *Pan* (Figure 2). For the DLW modeling, *H. erectus* would be 85% the cost of *A. afarensis*. However, the total cost of the reproductive event is lower. For *A. afarensis* modeled as *Pan* and *H. erectus* modeled as a human, the DLW‐modeled TEE is only 89.6% (*A. afarensis*: 3103 vs. 2779 Mcal) and 89.0% ( *H. erectus* : 2642 vs. 2365 Mcal) of the total TEE predicted by the A&K additive modeling. Under both modeling scenarios (A&K and DWL), if the large‐bodied *H. erectus* retained the more extended *Pan* lactation period and longer overall interbirth interval, its cost per reproductive event would be between 66.6% (DWL: 3940 vs. 2365 Mcal) and 78.6% (A&K: 4718 vs. 2642 Mcal) higher than an *H. erectus* with a human reproductive pattern (Figure 2).

As with the original A&K modeling, considerable uncertainties surround the new DLW modeling. It is also unclear whether the *Pan* model is applicable to *A. afarensis* and the human model to *H. erectus* . There is a variety of reproductive schedules across the great apes (van Schaik and Isler 2012) and the early hominins likely had a variety of schedules (Dean 2010). There is also no reason to assume that *H. erectus* would have a fully human reproductive pattern. It is more likely that a transition to this pattern occurred over the course of hominin evolution. However, the modeling illustrates the significant advantage of transitioning to the shorter human reproductive schedule for larger hominins. As with the original A&K model, not only is the energetic cost per offspring lower, but there is also the possibility of more offspring per female. The new DLW estimates for TEE do not change these original A&K conclusions.

## 3 Human Life History Patterns and Cooperation‐Based Social Organization

One of the main unanswered questions is why body size increased in the hominins, resulting in higher daily energy costs. The body‐size‐related costs would occur irrespective of whether the jump to the modern human DLW‐determined energetic grade was made simultaneously with the increase in body size in *H. erectus* . The costs would have been higher if it had. Over the years, several explanations have been proposed to account for the increase in hominin body size. These include, for example, thermoregulation and water balance (Ruff 1991; Wheeler 1992, 1993), locomotor efficiency (Pontzer 2017b), longevity and life history (Charnov and Berrigan 1993), and the ability of larger mothers to provide increased energy to the developing offspring. This last hypothesis is important because of the necessity to support larger‐brained infants.

What we do know about body size is that *H. erectus* has a larger average body size than the australopiths and early *Homo*, although there is a considerable variation in size in the earlier hominins. We also know that hominins with body sizes less than 40 kg (and statures less than 140 cm) disappear after 1.4 mya (except for some of the later small‐brained remnant species, e.g., *H. floresiensis*, and *H. naledi*) (Will et al. 2017). This may not be a coincidence (Grabowski 2016). Grabowski provides quantitative genetic evidence that brain and body size may have co‐evolved, particularly during the transition to *H. erectus* , and that selection for brain size alone may have been sufficient to drive an increase in both. The other correlates of larger body size may be just that. Locomotor and thermoregulatory efficiency, for example, were undoubtedly important for hominin evolution, survival, and dispersal, but may not have been central drivers of body size (or brain size) evolution.

An increase in brain size may provide other pieces in the puzzle of human evolution. It may not have been possible for a larger‐brained hominin to breed fast enough to replace population numbers. This is the Gray Ceiling Hypothesis (GCH) (Isler and van Schaik 2012). In primates, a larger residual brain mass is correlated with a longer residual gestation period, a longer residual lactation period, and a longer residual time to sexual maturity. This adds up to a decreased residual fertility level, which would eventually reduce the maximum population growth rate below viability. If hominins lived a great ape lifestyle under the constraints outlined by Isler and van Schaik (2012), the tipping point in brain size would be about 600–700 cm^3^. This is the brain size found in *H. habilis* and early *H. erectus* .

Isler and van Schaik's conclusions are similar to those of Aiello and Key (2002). They conclude that the only way to increase fertility is to shorten the lactation period and the interbirth interval. In other words, increasing brain size would incentivize the adoption of a more human‐like reproductive schedule. Otherwise, the population would be in danger of extinction. According to Isler and van Schaik (2012), this could have been achieved only through cooperative breeding and redistributing resources toward mothers and dependent infants. As with Aiello and Key, this transition would have had to be well underway by the time of *H. erectus* , beginning approximately 1.9 million years ago. Arguments based on body size and brain size converge, with the same conclusions centering on the necessity of intergroup cooperation and food sharing for survival. This evidence suggests that the period between 2.0 and 1.5 million years ago was a pivotal time for hominin evolution.

Isler and van Schaik and Aiello and Key are not alone in the more recent literature in suggesting the necessity of cooperative breeding and redistribution of resources toward mothers and dependent offspring, given both the increase in body size and brain size in the hominins (Burkart et al. 2014; Burkart et al. 2009; Kramer 2010; Robson and Wood 2008). The question is how the hominins did it. How did they acquire the resources to support the energetic demands of a large body, a large brain, a more compressed reproductive schedule, and support for dependent offspring? The standard explanation has been a pivot to a higher‐quality diet (Aiello and Wheeler 1995; Leonard and Robertson 1997; Leonard et al. 2003), which may have included cooking or other forms of food processing (e.g., pounding or cutting) (Carmody et al. 2011). Kraft et al. (2021) suggest that hunting and gathering notably boost daily energy returns. Based on the energetic analysis of foraging in modern hunters and gatherers, these authors argue that cooperative hunting and gathering simply provides more calories in less time than ape foraging behavior. It is a high‐risk, high‐reward subsistence strategy that is only possible due to correlated cooperation and intergenerational food sharing, including a division of labor. A hunting and gathering subsistence strategy increases the rate of energy acquisition and, in their interpretation, is of greater overall importance than energy‐saving strategies, such as bipedalism (Pontzer 2017b), a brain/gut trade‐off (Aiello and Wheeler 1995), or sophisticated tool use (Kraft et al. 2021). This strategy may also have been essential in enabling non‐reproductive females and males to acquire additional calories, reducing the energetic demand on reproductive females through food sharing. Again, it is interesting, and probably more than a coincidence, that there is increasing evidence of the exploitation of high‐quality, high‐risk animal‐based food in the *H. habilis* to *H. erectus* time period leading up to and including 2.0–1.5 million years ago.

An unanswered question is timing. Did the energetic demands of a large body size, a large brain, a more compressed reproductive schedule, and support for dependent offspring precede the transition to hunting and gathering, cooperation, and intergenerational food sharing, or did they follow? It is probably more likely that these aspects evolved in tandem, ratcheting up with the increase of both brain and body size through the evolution of *Homo*. More research is needed on this topic.

## 4 Conclusion

Although the energetic assumptions underlying the original Aiello and Key (2002) paper were incorrect, the general conclusions have withstood the test of time. Modeling based on doubly labeled water (DLW) estimates for TEE and modern assumptions about the costs of gestation and lactation results in the same conclusion. The adoption of a human reproductive schedule would have significantly lowered the energetic cost per offspring for a *H. erectus* female, irrespective of her increased body size. Where Aiello and Key (2002) underestimated TEE in relation to modern DLW predictions, they also significantly overestimated the costs of gestation and lactation. These two factors cancel each other out in the modeling, resulting in the same conclusion and highlighting the advantage of a modern human reproductive schedule.

It remains unclear when hominins adopted a modern reproductive schedule. The only real clue is from dental development, which suggests that the pattern of growth and development in australopiths and early *Homo* was consistent with modern apes (Dean et al. 2020; Dean 2010; Smith et al. 2015). In contrast, fully human growth and development patterns did not appear until modern *H. sapiens* . However, evidence about the constraints imposed by brain size increase (Isler and van Schaik 2012), the relationship between brain size increase and body size increase (Will et al. 2017), and the energetic advantages of a hunting and gathering lifestyle (Kraft et al. 2021) suggest that the transition was underway early in the evolution of the genus *Homo* and likely contributed to the success of *H. erectus* , its longevity as a species and its expansion out of Africa. This work also re‐emphasizes the fundamental importance of cooperation, food sharing, and high‐quality food in the evolution of humans.

## Ethics Statement

The author has nothing to report.

## Conflicts of Interest

The author declares no conflicts of interest.

## Data Availability

This is a modeling paper. There are no original data other than published body size estimates and energy equations.

## Endnotes

^1^
Total Energy Expenditure (TEE) is used in the DLW technique, while Daily Energy Expenditure (DEE) was used in the Aiello and Key (2002) modeling. They will be considered interchangeable for this discussion, and TEE will be used throughout.
